# Hypertriglyceridaemia-Associated Acute Pancreatitis: Pathophysiological Insights and Advances in Clinical Management

**DOI:** 10.3390/biomedicines14071574

**Published:** 2026-07-14

**Authors:** Yuying Li, Xinran Zhou, Yongjian Wen, Qianzi Huang, Xinmin Yang, Rajarshi Mukherjee, Robert Sutton, Tingting Liu, Wenhao Cai, Wei Huang

**Affiliations:** 1 West China Centre of Excellence for Pancreatitis, Institute of Integrated Traditional Chinese and Western Medicine, West China-Liverpool Biomedical Research Centre, West China Hospital, Sichuan University, Chengdu 610041, China; 2020224020178@stu.scu.edu.cn (Y.L.); claudia1204@163.com (Y.W.); huangqianzi@stu.scu.edu.cn (Q.H.); yangxinmin@wchscu.cn (X.Y.); liutingting@wchscu.cn (T.L.); 2 West China Biobank, West China Hospital, Sichuan University, Chengdu 610041, China; zhouxinran@wchscu.cn; 3 Liverpool Pancreatitis Research Group, Institute of Systems, Molecular and Integrative Biology, Liverpool University Hospitals NHS Foundation Trust, University of Liverpool, Liverpool L69 3GE, UK; rishim@liverpool.ac.uk (R.M.); r.sutton@liverpool.ac.uk (R.S.); 4 Department of Emergency General and Major Trauma Surgery, Aintree University Hospital, Liverpool University Hospitals NHS Foundation Trust, Liverpool L9 7AL, UK

**Keywords:** acute pancreatitis, hypertriglyceridaemia, aetiology, clinical management

## Abstract

Hypertriglyceridaemia (HTG) is an increasingly recognised aetiology of acute pancreatitis (AP), driven by the global rise in metabolic disorders. Although traditionally defined by triglyceride levels ≥ 1000 mg/dL, emerging evidence suggests a continuum of risk in which both genetic predisposition and secondary metabolic stressors contribute to disease onset and severity. Mechanistically, HTG-AP is characterised by lipotoxic injury mediated by free fatty acids, microcirculatory dysfunction, and amplified inflammatory responses, resulting in increased risk of organ failure and recurrence compared with AP of other aetiologies. Recent guideline updates emphasise early risk stratification, goal-directed supportive care, and prompt identification of underlying causes; however, HTG-specific management strategies remain heterogeneous. While insulin therapy and extracorporeal lipid removal techniques (e.g., plasmapheresis) are widely used, high-quality evidence supporting their superiority is limited. Long-term prevention relies on aggressive lipid control and modification of metabolic risk factors. This review provides a comprehensive synthesis of the aetiology, pathophysiology, clinical characteristics, and management of HTG-AP, highlighting recent advances, ongoing controversies, and future directions towards precision medicine.

## 1. Introduction

Acute pancreatitis (AP) is a leading cause of gastrointestinal-related hospitalisation worldwide, with incidence rising and a substantial healthcare burden [1,2]. Contemporary epidemiological data indicate that metabolic factors, including obesity, insulin resistance, and dyslipidaemia, are increasingly contributing to disease incidence, paralleling global lifestyle transitions [3]. Among these, hypertriglyceridaemia (HTG) has emerged as the third most common aetiology of AP, following biliary and alcohol-related causes [4,5]. Notably, in certain regions, particularly Eastern countries such as China, HTG has become the leading aetiological factor, reflecting the growing impact of metabolic disease on the epidemiology of AP [6,7,8,9,10].

HTG-associated AP (HTG-AP) is clinically distinct. Compared with other aetiologies, patients tend to be younger, more frequently male, and metabolically burdened, often presenting with obesity [11], diabetes mellitus (DM) [12], or metabolic syndrome [13]. Importantly, HTG-AP is also associated with greater disease severity, higher rates of persistent organ failure, and increased recurrence risk, suggesting that HTG is not merely a trigger but may act as a disease modifier that amplifies pancreatic injury and systemic inflammation [7,14,15].

Recent guideline updates and contemporary reviews emphasise early risk stratification, moderately aggressive goal-directed resuscitation, early enteral/oral nutrition when tolerated, and timely identification of disease aetiology as central pillars of AP management [4,16,17]. However, HTG-specific pathways are not yet fully integrated into these frameworks, reflecting ongoing uncertainty regarding causality thresholds, optimal triglyceride (TG) targets, and the role of extracorporeal therapies [18,19].

Previous reviews and systematic analyses have substantially advanced our understanding of HTG-AP by summarising individual aspects of its epidemiology, pathogenesis, clinical characteristics, and treatment [20,21,22,23]. However, updates to the framework that incorporate metabolic aetiology, lipid genetics, lipotoxic injury, microcirculatory impairment, immune activation, clinical phenotyping, risk stratification, and evidence-based management are still required. Accordingly, this review provides a clinically oriented and mechanistically integrated synthesis of HTG-AP, with particular emphasis on unresolved controversies, aetiology-specific risk stratification, the distinction between biochemical TG reduction and improving clinical outcomes, and future directions towards precision medicine.

## 2. Narrative Review Methodology

This narrative review was developed through structured searches of PubMed/MEDLINE, Embase, Web of Science, and the Cochrane Library for English-language articles from inception of the database to 30 June 2026, addressing hypertriglyceridaemia-associated acute pancreatitis, hyperlipidaemic acute pancreatitis, severe hypertriglyceridaemia, chylomicronaemia, triglyceride-rich lipoproteins, lipotoxicity, genetic susceptibility, risk stratification, insulin therapy, heparin, therapeutic plasma exchange, and emerging lipid-lowering therapies. Priority was given to clinical guidelines, systematic reviews, meta-analyses, prospective cohort studies, randomised trials, large retrospective studies, and mechanistic studies with direct relevance to HTG-AP. Additional references were identified from the bibliographies of selected articles.

## 3. Aetiology

Severe HTG, typically defined as TG levels ≥ 1000 mg/dL (≥11.3 mmol/L), is widely recognised as a causative factor for AP. However, the precise threshold for causality remains debated, as accumulating evidence suggests that the risk of AP increases progressively across a spectrum of TG levels, particularly in the presence of coexisting metabolic or genetic predispositions [19,24,25]. This has led to the concept of HTG functioning not only as a primary trigger but also as a disease modifier, lowering the threshold for pancreatic injury and amplifying inflammatory responses [26]. The aetiology of HTG-AP is broadly categorised into primary (genetic) and secondary (acquired) causes, which frequently interact within a “multiple-hit” framework (Table 1).

### 3.1. Primary Genetic Causes

The Fredrickson classification system categorises hyperlipoproteinaemia into six phenotypes based on lipoprotein profiles, among which Types I, IV, and V are most commonly associated with HTG and HTG-AP [27,28]. Although this classification remains clinically useful, recent advances in genomics have revealed substantial heterogeneity within these phenotypes.

#### 3.1.1. Type I Hyperlipoproteinaemia

Type I hyperlipoproteinaemia, also known as familial chylomicronaemia syndrome (FCS), is a rare autosomal recessive disorder characterised by near-complete deficiency of lipoprotein lipase (LPL) activity [29,30]. The majority of cases arise from mutations in the LPL gene, while less frequent causes include variants in genes encoding critical LPL cofactors or transport proteins, such as apolipoprotein C-II, apolipoprotein A-V, glycosylphosphatidylinositol-anchored high-density lipoprotein binding protein-1 (GPIHBP1), and lipase maturation factor-1 [30,31,32]. Clinically, FCS is characterised by persistent chylomicronaemia with markedly elevated TG levels, often exceeding 1500–2000 mg/dL [29,33,34], and recurrent episodes of pancreatitis beginning in childhood or adolescence [29]. Importantly, recent studies have identified autoantibodies against GPIHBP1 as a rare acquired mechanism that phenocopies FCS, thereby expanding the diagnostic spectrum beyond classical monogenic disorders [35]. From a pathophysiological perspective, FCS represents a “pure” model of TG-driven AP [36], providing key insights into the direct lipotoxic effects of chylomicron-derived free fatty acids (FFAs) and their role in acinar cell injury and microvascular dysfunction.

#### 3.1.2. Type IV Hyperlipoproteinaemia

Type IV hyperlipoproteinaemia (familial HTG) is a common polygenic disorder with an estimated prevalence of 5–10% [19]. It is characterised by increased hepatic production of very-low-density lipoprotein (VLDL), leading to moderate elevations in TG levels (typically 200–1000 mg/dL). In contrast to FCS, AP in familial HTG is uncommon unless TG levels rise into the severe range, usually in the presence of secondary exacerbating factors such as alcohol misuse, poorly controlled DM, or obesity [19]. This highlights the importance of gene–environment interactions in disease expression.

#### 3.1.3. Type V Hyperlipoproteinaemia

Type V hyperlipoproteinaemia is characterised by a combined elevation of chylomicrons and VLDL, reflecting a mixed dyslipidaemic phenotype. It is typically a polygenic disorder influenced by environmental and metabolic factors [37]. Patients often present in adulthood with severe HTG (frequently >886 mg/dL) and are at increased risk of both AP and atherosclerotic cardiovascular disease [34]. Elevated apolipoprotein B (ApoB) levels help distinguish this phenotype from FCS [29,38]. This condition exemplifies the interaction between inherited susceptibility and metabolic stressors, reinforcing the concept of HTG-AP as a multifactorial disease.

#### 3.1.4. Emerging Genetic Insights

Recent genomic and transcriptomic studies have refined our understanding of HTG by identifying both rare and common variants affecting lipid metabolism. In particular, genes involved in the regulation of LPL activity—such as angiopoietin-like proteins (ANGPTL) 3, 4, and 8—have emerged as key modulators of TG levels and potential therapeutic targets [39]. Polygenic risk scores incorporating multiple lipid-associated variants have demonstrated that cumulative genetic burden can significantly influence susceptibility to severe HTG and HTG-AP [40]. These findings support a “multiple-hit” model, in which genetic predisposition lowers the threshold for disease, while environmental or metabolic triggers determine clinical expression.

#### 3.1.5. Clinical Implications of Genetic Findings

Genetic findings may help guide lipid specialist referral, family counselling, pregnancy planning, medication avoidance, and recurrence prevention, but should be interpreted with clinical phenotyping since most adult HTG-AP is multifactorial and current polygenic risk scores remain insufficiently validated [41,42]. Therapeutically, this distinction has practical implications. Polygenic or multifactorial HTG-AP should be managed by correcting secondary and environmental triggers, such as diabetes, obesity, alcohol exposure, pregnancy-related factors, and TG-raising medications, together with conventional TG-lowering therapy. In contrast, monogenic chylomicronaemia may require more targeted treatment according to the underlying genetic defect and residual LPL activity. For example, patients with absent or minimal LPL activity often respond poorly to LPL-dependent therapies, whereas apolipoprotein C-III (ApoC3)-targeted therapies may reduce chylomicronaemia through partly LPL-independent mechanisms. Olezarsen has shown substantial TG-lowering efficacy in FCS [36] and has been approved for usage to reduce TG levels and AP risk in adults with severe HTG [43].

### 3.2. Secondary Causes

Secondary metabolic factors such as obesity, diabetes, alcohol intake, pregnancy, and medications are major determinants of HTG. However, these exposures do not act independently; rather, their phenotypic expression is strongly modulated by underlying genetic susceptibility, particularly polygenic variation in TG-rich lipoprotein (TRL) metabolism [37]. Therefore, secondary factors should be conceptualised as metabolic “triggers” acting upon a genetically determined baseline capacity for TG clearance, rather than as independent causes.

#### 3.2.1. Alcohol

Alcohol consumption exerts complex, dose-dependent effects on lipid metabolism and is a major secondary contributor to HTG-AP. Acute alcohol intake inhibits LPL activity, thereby impairing the clearance of TRLs and leading to transient elevations in circulating TG levels. In contrast, chronic alcohol consumption promotes hepatic VLDL synthesis and secretion through increased delivery of FFAs to the liver and activation of lipogenic pathways [25]. At the metabolic level, ethanol oxidation increases the hepatic NADH/NAD^+^ ratio, suppressing mitochondrial β-oxidation of fatty acids and favouring their esterification into TGs, thereby contributing to both hepatic steatosis and systemic HTG [44]. Alcohol may also exacerbate insulin resistance, further impairing lipid homeostasis [45]. In addition, alcohol can directly sensitise pancreatic acinar cells to injury by increasing oxidative stress, disrupting intracellular calcium signalling, and enhancing inflammatory responses [46,47,48], thereby lowering the threshold for AP in the presence of elevated TG levels. Importantly, alcohol often acts synergistically with other metabolic risk factors—such as obesity, DM, and genetic susceptibility—resulting in a markedly increased risk of HTG-AP [49].

#### 3.2.2. Metabolic Disorders

Obesity, DM, and non-alcoholic fatty liver disease (NAFLD) are the dominant drivers of secondary HTG in contemporary populations [34,50]; obesity is one of the most notable metabolic stressors contributing to HTG, with more than 80% of overweight or obese individuals exhibiting varying degrees of dyslipidaemia, including elevated TG levels [19,51,52]. Uncontrolled DM is another major contributor to severe HTG; in a cohort of patients with very severe HTG (TG ≥ 2000 mg/dL), approximately 74% of cases were associated with poorly controlled DM [53], while uncontrolled type 2 DM is frequently accompanied by elevated levels of VLDL and chylomicrons, reflecting profound disturbances in TG-rich lipoprotein metabolism [54]. More than half of children with NAFLD meet the intervention thresholds for dyslipidaemia, further highlighting the close relationship between metabolic dysfunction and HTG [55].

Obesity, adipose tissue dysfunction, NAFLD and HTG should be viewed as interconnected components of disordered TG metabolism. In metabolically healthy adipose tissue, excess energy can be buffered through safe TG storage. However, in obesity, adipocyte hypertrophy is accompanied by chronic low-grade inflammation, impaired adipokine secretion, and enhanced lipolysis, leading to excessive release of FFAs into the circulation [56]. These metabolic disturbances contribute to systemic insulin resistance, which further exacerbates hepatic VLDL overproduction and reduces peripheral TRL clearance, and altered lipid homeostasis [57]. Insulin resistance is one of the central mechanisms linking obesity, DM, and NAFLD to HTG [50,58]. Insulin resistance promotes increased fatty acid flux from adipose tissue to the liver, enhances hepatic de novo lipogenesis, impairs hepatic fatty acid oxidation, and alters microsomal TG transfer protein synthesis and ApoB degradation, collectively resulting in excessive VLDL secretion [59,60,61]. In addition, insulin normally suppresses hormone-sensitive lipase and TG lipase activity in adipose tissue; therefore, insulin deficiency or resistance leads to uncontrolled lipolysis and increased fatty acid delivery to the liver [62,63,64]. Insulin is also essential for LPL activity, with impaired TG clearance secondary to insulin deficiency being another important mechanism of HTG, particularly in type 1 DM [65,66]. Furthermore, diabetic ketoacidosis, a severe complication of DM characterised by profound insulin deficiency, is strongly associated with extreme HTG and increased risk of AP [67].

Collectively, these observations underscore the central role of metabolic dysregulation in the pathogenesis of HTG-AP and highlight key pathways that may serve as therapeutic targets.

#### 3.2.3. Medications

A range of medications have been implicated in the development of HTG and HTG-AP, including oestrogen-containing therapies, corticosteroids, protease inhibitors, and atypical antipsychotics [68]. These agents may elevate TG levels through multiple mechanisms, including increased hepatic lipid synthesis, reduced peripheral clearance of TRLs, and induction of insulin resistance [68]. Given their potential contribution to severe HTG, a comprehensive medication history and careful review of recent drug exposure are essential components of the clinical assessment in patients presenting with HTG-AP.

#### 3.2.4. Pregnancy

Pregnancy is associated with physiological hyperlipidaemia, driven by hormonal changes that increase hepatic lipogenesis and reduce LPL activity [69]. TG levels typically rise two- to three-fold during late gestation but rarely exceed 300 mg/dL in healthy individuals [70]. However, in the presence of underlying genetic or metabolic predispositions, TG levels may increase dramatically, leading to HTG-AP [70,71]. This condition is associated with significant maternal and foetal morbidity and requires multidisciplinary management, including obstetric, endocrinological, and critical care input [72].

## 4. Pathogenesis

**Figure 1:**
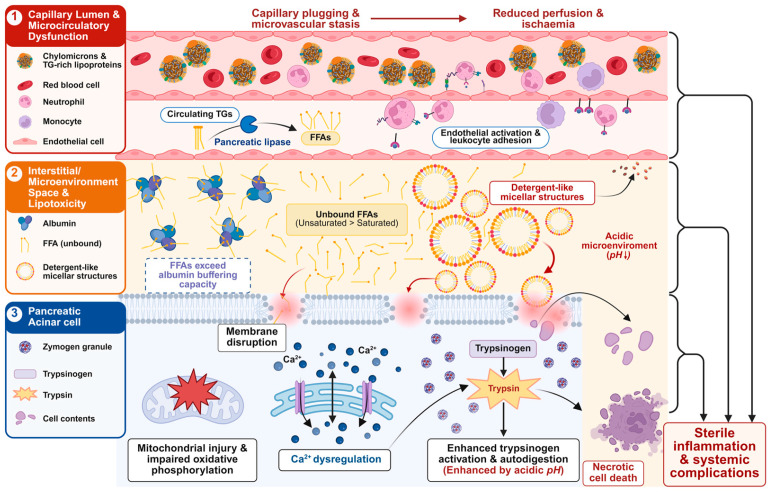
Pathogenesis of hypertriglyceridaemia-associated acute pancreatitis.

HTG-AP is best conceptualised as a lipotoxic–inflammatory disorder in which excess TG burden converges with the canonical pathways of AP [73]. In this framework, TRLs (particularly chylomicrons) provide a substrate for local lipolysis within the pancreatic microcirculation, generating toxic lipid intermediates that initiate acinar cell injury and amplify sterile inflammation (Figure 1). This model integrates metabolic overload with well-established mechanisms of AP, including intracellular calcium dysregulation, mitochondrial injury, and inflammatory signalling, thereby explaining the greater severity and systemic complications observed in HTG-AP [20,21,25,26].

### 4.1. Lipotoxicity and FFAs

A central pathogenic event in HTG-AP is the excessive hydrolysis of circulating TGs by pancreatic lipase, yielding high concentrations of FFAs [74,75,76]. When FFA levels exceed the buffering capacity of albumin, unbound FFAs exert direct cytotoxic effects on pancreatic acinar cells and pancreatic endothelial cells [77]. Mechanistically, these lipotoxic species disrupt cellular membranes, impair mitochondrial oxidative phosphorylation, and promote necrotic rather than apoptotic cell death. In addition, FFAs can form micellar structures with detergent-like properties that further damage cellular and vascular integrity. Local accumulation of FFAs also leads to the generation of an acidic microenvironment within pancreatic tissue, which enhances trypsinogen activation and potentiates enzymatic autodigestion [75]. Experimental models have demonstrated that unsaturated FFAs, particularly oleic acid and linoleic acid, exert stronger cytotoxic and pro-inflammatory effects than saturated fatty acids, highlighting the importance of lipid composition in modulating disease severity [78,79,80]. Beyond FFAs, other lipid species may also contribute to pancreatic injury; for example, lysophosphatidylethanolamine has been shown to induce acinar cell damage in vitro, suggesting that HTG-AP-related lipotoxicity may involve a broader spectrum of pathogenic lipid mediators [81].

### 4.2. Microcirculatory Dysfunction

HTG profoundly alters pancreatic microcirculation. Chylomicrons, which are markedly increased in severe HTG, are large lipoprotein particles that impede capillary blood flow, thereby increasing blood viscosity and promoting microvascular stasis [34]. This “capillary plugging” phenomenon results in reduced tissue perfusion, leading to pancreatic ischaemia and exacerbation of acinar cell injury [82]. Endothelial dysfunction further contributes to this process. Elevated FFAs and TRLs induce endothelial activation and injury, increasing vascular permeability and promoting leukocyte adhesion. In parallel, the dysregulation of vasoactive mediators, characterised by increased thromboxane A2 and reduced prostaglandin synthesis, favours vasoconstriction and worsens microvascular perfusion [20]. Collectively, these alterations create a hypoxic and pro-inflammatory milieu that accelerates necrosis and disease progression.

### 4.3. Inflammatory Amplification

Lipotoxic injury in HTG-AP triggers a complex immunometabolic inflammatory response [73,83]. FFAs act as danger-associated molecular patterns, triggering intracellular signalling cascades such as nuclear factor-κB and inflammasome activation [84,85]. This results in the release of pro-inflammatory cytokines, including interleukin-1β (IL-1β), tumour necrosis factor-α (TNF-α), and interleukin-6, which orchestrate both local pancreatic inflammation and systemic immune responses [20,86]. In addition, caspase-1, activated by Nod-like receptor family pyrin domain-containing 3, cleaves gasdermin D, triggering pore formation in the cell membrane and inducing pyroptotic cell death, which further exacerbates acinar cell injury and inflammatory propagation [87]. Activated neutrophils infiltrate the pancreas and exacerbate tissue injury through the release of reactive oxygen species, proteases, and neutrophil extracellular traps, contributing to endothelial dysfunction, microcirculatory impairment, and tissue necrosis [83,88]. In parallel, macrophages amplify inflammatory signalling via polarisation toward a pro-inflammatory M1 phenotype, characterised by increased secretion of TNF-α, IL-1β, and chemokines such as C-C motif chemokine ligand 2, thereby promoting further immune cell recruitment and sustaining a self-amplifying inflammatory loop. Impaired M2 polarisation may further delay inflammation resolution and prolong tissue injury [83]. In HTG-AP, the magnitude of this inflammatory response appears to be enhanced, likely reflecting the additive effects of metabolic and lipotoxic stress.

### 4.4. Calcium Overload and Cellular Stress

Consistent with the established mechanisms of AP, intracellular calcium dysregulation plays a pivotal role in HTG-AP [89]. Lipotoxic stress induces sustained elevations in cytosolic Ca^2+^ concentration, which disrupts mitochondrial function and promotes the opening of the mitochondrial permeability transition pore [90]. This leads to ATP depletion, oxidative stress, and cell death. In parallel, endoplasmic reticulum stress and unfolded protein responses are activated, further impairing cellular homeostasis and promoting inflammatory signalling [91]. These converging pathways ultimately drive acinar cell necrosis, which is a key determinant of severe disease and local complications such as pancreatic necrosis.

### 4.5. Integrative Perspective

Considering the above evidence, HTG-AP should be viewed as a metabolically primed form of AP in which excessive lipid burden lowers the threshold for pancreatic injury and amplifies downstream inflammatory cascades. This integrative model aligns with emerging concepts that AP severity is not solely determined by the initiating insult but is critically influenced by the host’s metabolic and immunological milieu [26,92]. Nevertheless, several mechanistic uncertainties remain. First, although circulating TG concentration is useful for diagnosis and clinical risk stratification, TG itself may not be the proximal injurious mediator; instead, TG-derived FFAs appear to play a more direct role in lipotoxic injury [73]. Second, the relative contribution of chylomicron-mediated microvascular plugging versus that of local lipase-mediated FFA generation remains unresolved. The limited clinical benefit of plasmapheresis despite rapid TG reduction suggests that relieving lipid obstructions in the circulation alone may not reverse established local lipotoxic injury; rather, chylomicrons may promote microvascular stasis and provide substrates for local lipolysis, whereas FFA generation determines the extent of cellular injury [73,83,93]. Third, lipotoxicity is likely to be lipid species-dependent, varying according to lipid class and molecular composition, including FFAs and other bioactive lipid intermediates [73,75]. Understanding HTG-AP through this lens provides a mechanistic rationale for targeted therapeutic strategies, including early TG reduction, modulation of lipotoxic pathways, and control of systemic metabolic dysfunction.

## 5. Diagnosis and Clinical Phenotype

### 5.1. Diagnostic Criteria

The diagnosis of HTG-AP is based on the standard criteria for AP, as defined by the Revised Atlanta Classification, which require the presence of at least two of the following three features: characteristic acute upper abdominal pain, serum amylase and/or lipase activity at least three times greater than the upper limit of normal, and imaging findings consistent with AP [94]. In the context of suspected HTG-AP, these criteria are supplemented by evidence of elevated serum TG levels and exclusion of alternative primary aetiologies, such as biliary or alcohol-related disease. Although a TG threshold of ≥1000 mg/dL (≥11.3 mmol/L) is most commonly used to define HTG as the causative factor, there is increasing recognition that lower TG levels, particularly in the range of 500–1000 mg/dL, may contribute to disease pathogenesis when combined with additional metabolic or genetic risk factors [7,15,19,25]. A single admission TG measurement may underestimate the true lipid burden in HTG-AP, as TG levels can decline rapidly following fasting, fluid resuscitation, insulin therapy, or inter-hospital transfer. Therefore, TG should be measured as early as possible in the disease course and interpreted alongside prior lipid profiles and the overall metabolic context to improve diagnostic accuracy [20,95]. HTG-AP frequently arises in mixed-aetiology settings, as severe HTG often coexists with other precipitating factors such as alcohol consumption, gallstones, pregnancy, or medication exposure [20]. In this context, HTG may function as a primary driver, a cofactor lowering the threshold for pancreatic injury, or a disease modifier, supporting a continuum-of-risk rather than a strict threshold-based model.

### 5.2. Laboratory Challenges

The diagnosis of HTG-AP presents unique biochemical challenges due to the interference of severe lipaemia with standard laboratory assays. Markedly elevated TG levels can result in spuriously low or normal serum amylase measurements, reducing the sensitivity of this marker for AP [20,96]. This phenomenon is attributed to analytical interference caused by lipaemic serum, which can be partially mitigated by serial dilution techniques. In contrast, serum lipase is less affected by lipaemia and is therefore considered a more reliable diagnostic biomarker in HTG-AP, occasionally misleading lipase values [97]. Additionally, severe hyperlipidaemia can lead to pseudohyponatraemia, a laboratory artefact resulting from displacement of the aqueous plasma fraction by excess lipids, leading to falsely low sodium concentrations when measured using indirect ion-selective electrodes [98]. Recognition of these artefacts is essential to avoid diagnostic errors and inappropriate clinical management.

### 5.3. Clinical Characteristics

HTG-AP exhibits a distinct clinical phenotype compared with AP of other aetiologies. Patients are more likely to present with more severe disease, characterised by higher rates of persistent organ failure, increased need for intensive care unit admission, and prolonged hospitalisation [7,15,99,100]. They also have increased risks of multiple organ dysfunction syndrome and mortality [101], recurrent AP [102], and long-term complications, including DM [7], exocrine pancreatic insufficiency [103], and chronic pancreatitis [14,104,105]. Furthermore, the increased risk of recurrent AP in HTG-AP is closely associated with poorly controlled TG levels and persistent metabolic dysfunction [106]. However, the relationship between serum TG levels and clinical outcomes requires careful interpretation. The prognostic value of admission TG concentration is inconsistent across studies, and a robust linear dose–response relationship between TG level and acute clinical outcomes has not been firmly established [95]. This inconsistency may be explained by differences in the timing of TG measurement relative to symptom onset, rapid TG decline after fasting and fluid resuscitation, heterogeneity in diagnostic thresholds for HTG-AP, and confounding by metabolic comorbidities, including obesity, diabetes, insulin resistance, and NAFLD. Therefore, admission TG levels should not be used as a stand-alone marker for predicting severity, organ failure, or mortality. Instead, they should be interpreted alongside dynamic TG changes, systemic inflammatory markers, organ dysfunction, imaging findings, and the broader metabolic context.

### 5.4. Risk Stratification

Risk stratification in HTG-AP should integrate conventional early assessment of AP severity with aetiology-specific metabolic and inflammatory features. Current guidelines emphasise early haemodynamic assessment, identification of systemic inflammatory response syndrome (SIRS) or organ failure, and close reassessment during the first 48 h, while recognising that scoring systems or imaging alone are insufficient to reliably predict progression to moderately severe or severe AP; elevated blood urea nitrogen, haematocrit, obesity, comorbidities, and SIRS remain practical early warning markers [4,17]. In HTG-AP, admission TG levels should not be interpreted in isolation. A 2025 systematic review including 77 studies and 56,617 AP patients, of whom 11,315 had HTG-AP, showed that HTG-AP patients were generally younger, predominantly male, and had higher rates of severe disease, mortality, and recurrence than non-HTG-AP patients; disease severity was associated not only with elevated TG levels, but also with pancreatic necrosis, SIRS, shock, multi-organ failure, high neutrophil-to-lymphocyte ratio, elevated C-reactive protein, hypocalcaemia, and hypoalbuminaemia [23]. A multicentre cohort study further demonstrated that HTG was independently associated with systemic and local complications of AP, including SIRS, shock, acute respiratory distress syndrome, acute renal failure, and necrotic collections, and developed a prediction model for non-mild AP that discriminated well between the derivation and validation cohorts [107]. More recent HTG-AP-specific models have incorporated computed tomography findings, fatty liver infiltration, and blood biomarkers to improve prediction of severe HTG-AP, although these tools still require broader prospective validation before routine clinical implementation [108]. Dynamic metabolic monitoring may also be clinically relevant, as a 2024 propensity-score-matched study found that admission TG levels were not directly correlated with HTG-AP severity, whereas achieving TG reduction below 5.56 mmol/L within 48–72 h was associated with fewer local complications and persistent organ failure [109]. Therefore, high-risk HTG-AP should be suspected in patients with early SIRS or organ dysfunction, obesity, diabetes, fatty liver, marked inflammatory responses, hypocalcaemia, hypoalbuminaemia, rising blood urea nitrogen or haematocrit, or delayed TG clearance, and these patients require close monitoring, early organ-support assessment and structured prevention of recurrence. Furthermore, comorbid metabolic disorders (e.g., fatty liver disease [110,111]), body composition [112], admission glucose levels [113,114,115], and detailed lipid profiles (e.g., apolipoprotein A-I [112], TG/high-density lipoprotein ratio [116,117]) may provide additional value for predicting disease severity.

Conventional AP severity scores have been evaluated in HTG-AP populations, but their performance appears to be outcome-specific. In a retrospective Chinese cohort of 326 patients with HTG-AP, Bedside Index for Severity in Acute Pancreatitis (BISAP), Ranson, modified computed tomography severity index (MCTSI), and Acute Physiology and Chronic Health Evaluation II (APACHE II) were all associated with disease severity, local complications and mortality; APACHE II showed the best discrimination for predicting moderately severe or severe disease, MCTSI performed best for local complications, and BISAP had the highest area under the curve for mortality prediction while being simpler to apply [118]. However, these tools were developed for AP in general and do not incorporate HTG-AP-specific metabolic features, such as TG burden, dynamic TG clearance, insulin resistance, fatty liver, or lipid-derived indices. Therefore, conventional scores remain useful for early clinical assessment but should be interpreted within a broader HTG-AP-specific risk-stratification framework integrating organ dysfunction, systemic inflammation, metabolic comorbidities, TG-related parameters, and imaging findings. Table 2 summarises the current evidence on risk stratification for HTG-AP.

## 6. Management

### 6.1. General Management

**Figure 2:**
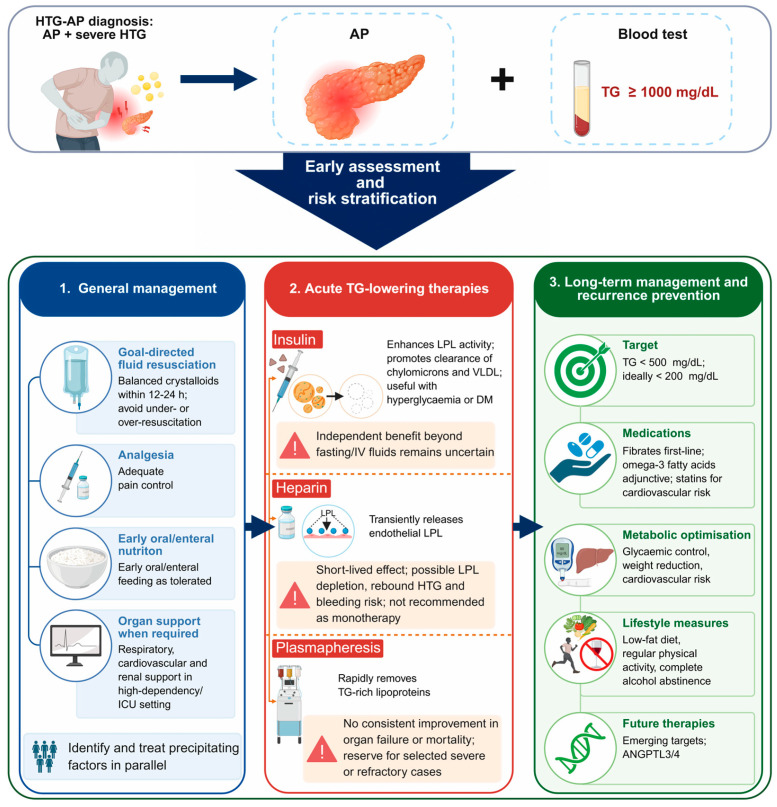
Management of hypertriglyceridaemia-associated acute pancreatitis.

General supportive care remains the cornerstone of management for HTG-AP and is supported by high-quality evidence and international guideline consensus across all AP aetiologies, including guidance from the American College of Gastroenterology, International Association of Pancreatology, and World Society of Emergency Surgery [4,17,123]. Early, goal-directed fluid resuscitation, preferably with balanced crystalloids, is recommended within the first 12–24 h, using careful titration to avoid both hypovolaemia and fluid overload, as excessive resuscitation has been associated with worse outcomes [124]. Adequate analgesia, typically with opioid-based regimens, is essential for symptom control and to reduce physiological stress. Early nutritional support is now a cornerstone of AP management, with initiation of oral or enteral feeding as soon as tolerated, even in moderately severe disease, to preserve gut barrier integrity and reduce infectious complications. In patients who develop organ failure, management should follow a multidisciplinary, organ-supportive approach, including respiratory, cardiovascular, and renal support where indicated, ideally in a high-dependency or intensive care setting [5,123]. Importantly, early identification and treatment of precipitating factors, including severe HTG, should be undertaken in parallel with supportive care. The management framework for HTG-AP is illustrated in Figure 2.

### 6.2. Acute TG-Lowering Therapies

In HTG-AP, a rapid reduction in circulating TG levels is a rational therapeutic target, given the central role of TRLs in disease pathogenesis [125]. However, the optimal approach to acute TG reduction remains an area of ongoing debate, as high-quality randomised evidence is limited [126]. Insulin, heparin, and plasmapheresis can all reduce serum TG levels in HTG-AP, but none have demonstrated clear superiority in improving patient-centred outcomes such as mortality, persistent organ failure, or local complications. The choice of therapy is therefore driven primarily by disease severity, resource availability, and safety considerations.

#### 6.2.1. Insulin

Insulin therapy is supported by moderate-quality evidence and is widely used, particularly in patients with concurrent hyperglycaemia or DM [4]. Insulin enhances the synthesis and activity of LPL, thereby accelerating the clearance of chylomicrons and VLDL, and reduces hepatic TG production by suppressing lipolysis in adipose tissue [65,127]. In addition to its lipid-lowering effects, insulin may confer cytoprotective benefits by attenuating lipotoxic injury, stabilising intracellular calcium homeostasis, and improving metabolic derangements associated with AP [19,128,129]. Additionally, disturbances in glucose metabolism are not only closely linked to TG metabolism but are also independently associated with worse clinical outcomes in AP. Emerging evidence suggests that insulin therapy may improve outcomes in HTG-AP, partly through reducing glycaemic variability and improving metabolic homeostasis [114,130]. Moreover, intensive insulin therapy has been reported to shorten hospital stay and reduce Acute Physiology and Chronic Health Evaluation II (APACHE II) scores after 72 h of treatment in patients with severe AP [131]. However, a retrospective study comparing insulin therapy with conservative management alone reported that insulin did not accelerate TG reduction in patients with HTG-AP compared with supportive treatment alone [132]; fasting and intravenous fluid resuscitation were highly effective in rapidly lowering concentrations of circulating TG, with insulin providing no additional benefit in the rate of reducing TG. Consequently, although insulin therapy remains widely adopted in clinical practice, its independent therapeutic benefit beyond supportive care requires further validation in prospective randomised studies.

#### 6.2.2. Heparin

Heparin has historically been used as an adjunctive therapy due to its ability to transiently release LPL from endothelial binding sites into the circulation, thereby enhancing TG clearance. However, this effect is short-lived, as continuous heparin exposure accelerates hepatic degradation of LPL, potentially leading to depletion of enzyme stores and rebound HTG [133,134]. Accordingly, only short-term use may be considered in selected cases of HTG-AP [4]. Recent clinical evidence regarding the efficacy of heparin in HTG-AP has been conflicting. In a large retrospective cohort study involving 2862 patients with HTG-AP, early administration of low-molecular-weight heparin (LMWH) was associated with reduced in-hospital mortality, lower risk of persistent organ failure, and decreased need for renal replacement therapy without increasing bleeding complications [135]. In contrast, a multicentre randomised trial conducted by the same study group, which included 533 patients with HTG-AP, demonstrated that the addition of LMWH to insulin therapy did not improve organ failure, mortality, or TG reduction compared with insulin alone [136]. These findings suggest that the routine addition of heparin may not provide additional benefits to early lipid-lowering therapy in HTG-AP. Moreover, given the potential risk of bleeding, particularly in patients with pancreatic necrosis or severe disease, the routine use of heparin remains controversial and is generally not recommended as a monotherapy [20].

#### 6.2.3. Plasmapheresis

Plasmapheresis provides a rapid and effective method of lowering circulating TG levels by directly removing TRLs from the plasma [137,138,139,140]. However, its effect on clinical outcomes remains unproven, and the overall quality of evidence is low. It may be considered in patients with persistent organ failure and markedly elevated TG levels, particularly when complicated by acute renal failure [4]. Several observational studies and small series have demonstrated substantial reductions in TG levels within 24–48 h, along with improvements in biochemical and inflammatory markers [137,138]. However, the current evidence does not consistently demonstrate a clear benefit in clinically meaningful outcomes, such as reduced organ failure, complications, or mortality, when compared with conservative management [18,139]. Recent multicentre prospective studies have questioned the clinical benefit of plasmapheresis in HTG-AP, demonstrating that although plasmapheresis effectively lowers circulating TG levels, it was not associated with reduced organ failure incidence or duration, or improved mortality after adjustment for confounders, and was consistently associated with increased intensive care unit admission requirements compared with medical treatment alone [93,141]. Given its invasiveness, cost, and limited availability, plasmapheresis is generally reserved for selected patients with severe or refractory HTG-AP, particularly those with extremely elevated TG levels or inadequate response to medical therapy. A multicentre prospective cohort study demonstrated substantial variation in TG-lowering strategies across centres in HTG-AP; it was found that more rapid TG reduction was not associated with improved organ-failure-free days or reduced duration of organ failure, suggesting that aggressive early TG reduction may not necessarily translate into better clinical outcomes [142]. Overall, current studies, including observational cohorts and a small randomised trial, have not demonstrated a consistent benefit of plasmapheresis in improving organ failure, mortality, or disease severity compared with conservative therapy.

### 6.3. Long-Term Management

Long-term management of HTG-AP is primarily focused on preventing recurrence through sustained control of TG levels and correction of underlying metabolic disturbances. Serum TG concentrations should be maintained below 500 mg/dL (5.6 mmol/L) to reduce the risk of recurrent pancreatitis, with some data suggesting that levels below 200 mg/dL may provide additional protection [4,25]. Pharmacological therapy typically includes fibrates as first-line agents, given their efficacy in reducing TG levels through activation of peroxisome proliferator-activated receptor-α. Omega-3 fatty acids may be used as an adjunctive therapy, while statins can be considered in patients with coexisting cardiovascular risk, although their TG-lowering effects are modest [19]. Emerging therapies targeting key regulators of lipid metabolism, such as ANGPTL3/4, represent promising future options [39]. Equally important is the optimisation of metabolic comorbidities, including strict glycaemic control in DM, weight reduction in obesity, and management of NAFLD [143]. Lifestyle interventions comprising dietary fat restriction, regular physical activity, and complete abstinence from alcohol are essential components of long-term prevention of HTG-AP [19,21]. Taken together, effective management of HTG-AP requires a dual approach, combining early supportive care and targeted lipid-lowering strategies in the acute phase with sustained metabolic control to prevent recurrence and long-term complications.

## 7. Future Directions

Despite substantial advances in the understanding of HTG-AP, several critical gaps remain that limit the development of evidence-based, aetiology-specific management strategies. A key priority is to refine the definition of TG thresholds for causality. Future research should prioritise three areas with direct implications for patient care: well-designed randomised controlled trials of acute TG-lowering strategies, validation of HTG-specific risk prediction tools, and rigorous evaluation of emerging lipid-lowering therapies for prevention of recurrent pancreatitis and long-term complications.

First, adequately powered multicentre randomised controlled trials are urgently needed to determine whether rapid TG reduction improves clinical outcomes. Although both insulin therapy and therapeutic plasma exchange are commonly used in acute HTG-AP, current evidence remains limited and inconsistent. Future trials should not use TG reduction alone as the primary endpoint; instead, they should assess organ-failure-free days, persistent organ failure, intensive care unit admission, pancreatic necrosis, mortality, recurrence, adverse events, cost-effectiveness, and patient selection according to baseline TG level, diabetes or diabetic ketoacidosis, early organ dysfunction, and suspected monogenic versus multifactorial HTG.

HTG-specific risk prediction requires prospective validation. Conventional AP scoring systems were not developed specifically for HTG-AP and may not capture the metabolic drivers of disease progression. Therefore, there is increasing interest in incorporating metabolic biomarkers into risk prediction models. Traditional scoring systems for AP focus primarily on clinical and organ failure parameters; however, emerging evidence suggests that metabolic indicators, including TG levels, glycaemic variability, and markers of insulin resistance, may provide incremental prognostic value [15,114]. The incorporation of such biomarkers into dynamic prediction tools could improve early identification of high-risk patients and facilitate more targeted interventions. Future studies should test calibration, external validity, clinical utility, and decision impact across different populations before these tools are incorporated into routine practice.

Therapeutic innovation is also rapidly evolving. ApoC3 raises circulating TG levels by inhibiting LPL-mediated hydrolysis and the hepatic clearance of TG-rich remnants [144,145]. Volanesorsen, a first-generation APOC3 antisense oligonucleotide, substantially reduced TG levels in patients with FCS, but its use is limited by thrombocytopenia and injection-site reactions [146]. Olezarsen, a GalNAc-conjugated APOC3 antisense oligonucleotide, is effective in FCS and is the first FDA-approved therapy shown to reduce AP risk in adults with severe HTG [36,43,147]. Plozasiran, an APOC3-targeted small interfering RNA, also reduces TG levels in severe HTG and persistent chylomicronaemia, with emerging evidence of fewer pancreatitis events [148,149]. ANGPTL3, -4 and -8 are key regulators of TRL metabolism and potential therapeutic targets for severe HTG and recurrent HTG-AP [150,151,152]. All three inhibit LPL-mediated TG clearance, although their biological roles and therapeutic implications differ. ANGPTL3, mainly produced by the liver, inhibits LPL and endothelial lipase and has the most advanced clinical development [150,153]. Evinacumab, an ANGPTL3 monoclonal antibody, lowers TG levels in severe HTG, but responses vary with residual LPL activity and are likely limited in completely LPL-deficient FCS [150]. Hepatic ANGPTL3 silencing with RNA-based therapies also lowers atherogenic lipids [5]. However, vupanorsen was discontinued because of dose-dependent hepatic fat accumulation and elevated transaminase, highlighting the need to assess hepatic safety carefully, especially in patients with obesity, diabetes, or NAFLD [154,155]. ANGPTL4 is a more complex therapeutic target. Besides inhibiting LPL and modulating TG homeostasis, it may directly contribute to pancreatitis-associated inflammation. Experimental and human studies suggest that ANGPTL4 is increased in pancreatitis and may exacerbate disease through macrophage activation [156]. However, systemic ANGPTL4 blockades have raised safety concerns, including lymphatic lipid accumulation and inflammation in animal models, although newer antibody-based approaches suggest that safer inhibition may be feasible [157,158]. ANGPTL8 and the ANGPTL3/8 complex represent another emerging regulatory pathway. ANGPTL8 enhances ANGPTL3-mediated LPL inhibition in the fed state. Selective targeting of the ANGPTL3/8 complex may therefore improve TG-rich lipoprotein clearance while avoiding some effects of broad ANGPTL3 inhibition [152,159]. Overall, APOC3 inhibition currently has the strongest direct evidence for reducing TG and pancreatitis risk in severe HTG and FCS. Therapies targeting ANGPTL3-, ANGPTL4-, and ANGPTL3/8 remain promising but are still investigational and may depend on patient phenotype. However, most evidence for these emerging therapies comes from small or early-stage studies. Well-designed prospective clinical trials are therefore needed to determine their effects on recurrent pancreatitis, pancreatitis-related hospitalisation, organ failure, quality of life, and long-term hepatic and metabolic safety rather than relying solely on short-term TG reduction.

Collectively, these research priorities underscore the need for a multidimensional approach to HTG-AP, integrating mechanistic insights with clinical and translational innovation. Large-scale, prospective studies and randomised controlled trials will be critical to validate emerging concepts and translate them into clinical practice.

## 8. Conclusions

HTG-AP is an increasingly prevalent and clinically significant form of AP characterised by distinct metabolic and inflammatory mechanisms. Mounting evidence supports the central roles of lipotoxicity, microcirculatory dysfunction, and amplified immune responses in driving disease severity and systemic complications. Although advances in mechanistic understanding have improved clinical recognition and informed current management strategies, high-quality evidence guiding aetiology-specific interventions remains limited, and many aspects of care continue to rely on extrapolation from general AP guidelines. The evidence base for HTG-specific management remains incomplete. Key uncertainties include the TG threshold at which HTG is causal rather than contributory, the optimal acute TG-lowering strategy, the appropriate role and timing of insulin and plasmapheresis, the validity of HTG-specific prognostic tools, and the real-world effectiveness of emerging lipid-lowering therapies in preventing recurrent pancreatitis. Future progress in treating HTG-AP will depend on the adoption of precision medicine approaches, integrating genetic predisposition, metabolic context, and dynamic clinical parameters to refine diagnosis, risk stratification, and treatment. In particular, the development of targeted lipid-lowering therapies, improved phenotypic classification, and incorporation of metabolic biomarkers into clinical decision-making frameworks hold promise for transforming patient care. Ultimately, bridging the gap between mechanistic insight and clinical applications will be essential for optimising the outcomes and reducing the burden of this increasingly prevalent disease.

## Figures and Tables

**Table 1:** Aetiological factors of HTG-AP.

Category	Subtype/Factor	Key Characteristics
General concept	Severe HTG	TG ≥ 1000 mg/dL is widely recognised as a causative factor for AP, although the exact threshold remains debated
Primary genetic causes	Fredrickson classification	Types I, IV and V hyperlipoproteinaemia are most closely associated with HTG and HTG-AP
	Type I hyperlipoproteinaemia/FCS	Rare autosomal recessive disorder with near-complete LPL deficiency; TG often >1500–2000 mg/dL; recurrent AP from childhood or adolescence
	GPIHBP1 autoantibody-associated chylomicronaemia	Acquired condition that phenocopies FCS
	Type IV hyperlipoproteinaemia/familial HTG	Common polygenic disorder; TG typically 200–1000 mg/dL
	Type V hyperlipoproteinaemia	Mixed dyslipidaemic phenotype with elevated chylomicrons and VLDL; often presents in adulthood; TG frequently >886 mg/dL
Emerging genetic insights	ANGPTL3, ANGPTL4, ANGPTL8 and polygenic risk	Genetic variants affecting LPL regulation and TG metabolism
Secondary acquired causes	Alcohol	Acute alcohol intake inhibits LPL activity; chronic intake promotes hepatic VLDL synthesis
	Obesity	One of the most important metabolic stressors for HTG; dyslipidaemia is common in overweight or obese individuals
	DM	Poorly controlled DM is strongly associated with very severe HTG; DKA may cause extreme TG elevation
	NAFLD	Frequently associated with dyslipidaemia, including elevated TG levels
	Insulin resistance	Central mechanism linking obesity, DM and NAFLD to HTG
	Medications	Oestrogen-containing therapies, corticosteroids, protease inhibitors and atypical antipsychotics are commonly implicated
	Pregnancy	Physiological hyperlipidaemia occurs due to hormonal changes; TG rises two- to three-fold in late gestation

Abbreviations: HTG, hypertriglyceridaemia; TG, triglyceride; AP, acute pancreatitis; HTG-AP, hypertriglyceridaemia-associated acute pancreatitis; FCS, familial chylomicronaemia syndrome; LPL, lipoprotein lipase; GPIHBP1, glycosylphosphatidylinositol-anchored high-density lipoprotein-binding protein 1; VLDL, very low-density lipoprotein; ANGPTL, angiopoietin-like protein; DM, diabetes mellitus; DKA, diabetic ketoacidosis; NAFLD, non-alcoholic fatty liver disease.

**Table 2:** Performance of different indicators for risk stratification of HTG-AP.

Indicator/Model	Components or Key Variables	Timing	Outcome Predicted	Reported Performance	Reference (PMID)
Severe HTG-AP prediction model based on laboratory and imaging features	CRP, LDH, serum Ca^2+^, ascites	Within 24 h of admission	Severe HTG-AP	Model AUC 0.960; individual AUCs: LDH 0.893, CRP 0.886, Ca^2+^ 0.872, ascites 0.850	[119]
Nomogram based on CT findings and blood biomarkers	Serum calcium, CRP, LDH, liver-to-spleen CT attenuation ratio	Early admission with CT assessment	Severe HTG-AP	Training AUC 0.957; external validation AUC 0.930; sensitivity 91.3%, specificity 88.6% in training cohort	[108]
Early laboratory prediction model	CRP, D-dimer, RDW/SC	Early admission	Severe HTG-AP	Model AUC 0.915; sensitivity 91.9%, specificity 79.1%; individual AUCs: D-dimer 0.874, RDW/SC 0.843, CRP 0.831	[120]
Metabolic-index nomogram	LDH, serum creatinine, VATI, albumin, TyG index	Early admission plus CT body-composition assessment	Severe HTG-AP	Apparent AUC 0.966; internally validated average AUC 0.891; individual AUCs: LDH 0.825, Scr 0.821, albumin 0.808, VATI 0.637, TyG 0.635	[121]
Conventional AP scoring systems	APACHE II, BISAP, Ranson, MCTSI	Within 24–48 h; MCTSI requires imaging	Severity, local complications, mortality	APACHE II showed the best AUC for moderately severe AP/severe AP prediction 0.814; BISAP 0.795; Ranson 0.766; MCTSI 0.654. For mortality, BISAP AUC was 0.867 and Ranson 0.842	[118]
TG/HDL-C ratio	Triglyceride-to-high-density lipoprotein cholesterol ratio	Early admission	Severe HTG-AP	Independent predictor of severity; AUC 0.727, outperforming several conventional scoring systems in this cohort	[116]
Metabolic and lipid indices	TyG index, FIB-4 score, haemoglobin, TG/HDL-C ratio, TG/LDL-C ratio	Early admission; patients admitted within 48 h of abdominal pain onset	Severe HTG-AP	TG/HDL-C showed a nonlinear association with severe AP risk; TyG > 9.90, FIB-4 > 1.54, haemoglobin ≥ 143 g/L and TG/LDL-C > 21 were independently associated with severe AP	[117]
Body composition and laboratory markers	CT-based body composition parameters, serum albumin, CRP, TG	Admission; CT-based body composition assessment at L3 level	Moderately severe to severe HTG-AP	Low albumin < 35 g/L and CRP > 90 mg/L were independently associated with severity; albumin AUC 0.759 and CRP AUC 0.786, whereas CT-based body composition parameters were not associated with severity	[112]
Dynamic TG reduction	TG ≤ 5.65 mmol/L by 48–72 h or day 3	Serial monitoring during early treatment	Persistent organ failure or organ failure-free days	Earlier retrospective data suggested TG 48 h after admission ≥ 5.65 mmol/L was associated with persistent organ failure; however, another prospective cohort found that reaching TG ≤ 5.65 mmol/L by day 3 or faster TG decline was not associated with improved organ failure-free days	[109,122]
Admission TG level/HTG grade	TG concentration at presentation	Admission	Severity, necrosis, SIRS, organ failure, recurrence	Associations with severity have been reported, but predictive performance is inconsistent and no robust single cut-off reliably stratifies severity	[7]
Inflammatory and biochemical markers	CRP, NLR, hypocalcaemia, hypoalbuminaemia, BUN, haematocrit	Admission and repeated within 24–48 h	Severe disease, SIRS, organ failure	Individual performance varies across studies; systematic review data support their association with HTG-AP severity but not always with validated cut-offs	[23]

Abbreviations: AUC, area under the curve; HTG-AP, hypertriglyceridaemia-associated acute pancreatitis; CRP, C-reactive protein; LDH, lactate dehydrogenase; CT, computed tomography; AP, acute pancreatitis; RDW/SC, red blood cell distribution width-to-serum calcium ratio; VATI, visceral adipose tissue index; TyG, triglyceride-glucose index; Scr, serum creatinine; APACHE II, Acute Physiology and Chronic Health Evaluation II; BISAP, Bedside Index for Severity in Acute Pancreatitis; MCTSI, modified computed tomography severity index; TG, triglyceride; SIRS, systemic inflammatory response syndrome; NLR, neutrophil-to-lymphocyte ratio; BUN, blood urea nitrogen.

## Data Availability

No new data were created or analyzed in this study. Data sharing is not applicable to this article.

## Abbreviations

The following abbreviations are used in this manuscript:

ANGPTL	Angiopoietin-like protein
APACHE II	Acute Physiology and Chronic Health Evaluation II
ApoA5	Apolipoprotein A-V
ApoB	Apolipoprotein B
AP	Acute pancreatitis
AUC	Area under the curve
BISAP	Bedside Index for Severity in Acute Pancreatitis
BUN	Blood urea nitrogen
CRP	C-reactive protein
CT	Computed tomography
DKA	Diabetic ketoacidosis
DM	Diabetes mellitus
FFAs	Free fatty acids
FCS	Familial chylomicronaemia syndrome
GPIHBP1	Glycosylphosphatidylinositol-anchored high-density lipoprotein binding protein 1
HTG	Hypertriglyceridaemia
HTG-AP	Hypertriglyceridaemia-associated acute pancreatitis
IL-1β	Interleukin-1β
LDH	Lactate dehydrogenase
LPL	Lipoprotein lipase
LMWH	Low-molecular-weight heparin
MCTSI	Modified computed tomography severity index
NAFLD	Non-alcoholic fatty liver disease
NLR	Neutrophil-to-lymphocyte ratio
RDW/SC	Red blood cell distribution width-to-serum calcium ratio
ROC	Receiver operating characteristic
Scr	Serum creatinine
SIRS	Systemic inflammatory response syndrome
SOFA	Sequential Organ Failure Assessment
TG	Triglyceride
TNF-α	Tumour necrosis factor-α
TRL	Triglyceride-rich lipoprotein
TyG	Triglyceride-glucose index
VATI	Visceral adipose tissue index
VLDL	Very low-density lipoprotein

## References

[1] Petrov M.S., Yadav D. (2019). Global epidemiology and holistic prevention of pancreatitis. Nat. Rev. Gastroenterol. Hepatol..

[2] Iannuzzi J.P., King J.A., Leong J.H., Quan J., Windsor J.W., Tanyingoh D., Coward S., Forbes N., Heitman S.J., Shaheen A.A. (2022). Global Incidence of Acute Pancreatitis Is Increasing over Time: A Systematic Review and Meta-Analysis. Gastroenterology.

[3] Petrov M.S., Olesen S.S. (2023). Metabolic Sequelae: The Pancreatitis Zeitgeist of the 21st Century. Gastroenterology.

[4] Párniczky A., Mikó A., Uc A., Singh A.N., Elhence A., Saluja A., Masamune A., Dayyeh B.K.A., Davidson B., Wilcox C.M. (2025). International Association of Pancreatology Revised Guidelines on Acute Pancreatitis 2025: Supported and Endorsed by the American Pancreatic Association, European Pancreatic Club, Indian Pancreas Club, and Japan Pancreas Society. Pancreatology.

[5] Szatmary P., Grammatikopoulos T., Cai W., Huang W., Mukherjee R., Halloran C., Beyer G., Sutton R. (2022). Acute Pancreatitis: Diagnosis and Treatment. Drugs.

[6] Ding Y., Zhang M., Wang L., Yin T., Wang N., Wu J., Zhi J., Chen W., Wu K., Gong W. (2019). Association of the hypertriglyceridemic waist phenotype and severity of acute pancreatitis. Lipids Health Dis..

[7] Zhang R., Deng L., Jin T., Zhu P., Shi N., Jiang K., Li L., Yang X., Guo J., Yang X. (2019). Hypertriglyceridaemia-associated acute pancreatitis: Diagnosis and impact on severity. HPB.

[8] Shi N., Liu T., de la Iglesia-Garcia D., Deng L., Jin T., Lan L., Zhu P., Hu W., Zhou Z., Singh V. (2020). Duration of organ failure impacts mortality in acute pancreatitis. Gut.

[9] Ke L., Zhou J., Mao W., Chen T., Zhu Y., Pan X., Mei H., Singh V., Buxbaum J., Doig G. (2022). Immune enhancement in patients with predicted severe acute necrotising pancreatitis: A multicentre double-blind randomised controlled trial. Intensive Care Med..

[10] Lai T., Li J., Zhou Z., Rao J., Zhu Y., Xia L., Lei Y., Huang X., Ke H., Wu Y. (2024). Etiological Changes and Prognosis of Hospitalized Patients with Acute Pancreatitis over a 15-Year Period. Dig. Dis. Sci..

[11] Khatua B., El-Kurdi B., Singh V.P. (2017). Obesity and pancreatitis. Curr. Opin. Gastroenterol..

[12] Dungan K.M., Chinchilli V.M., Pichardo-Lowden A., Raja-Khan N., Bellin M.D., Yadav D., Hart P.A., Basina M., Buxbaum J.L., Casu A. (2026). Hyperglycemia During Acute Pancreatitis and Progression to Early-Onset Diabetes After Recovery: Preliminary Findings from the Diabetes Related to Acute Pancreatitis and Its Mechanisms (DREAM) Study. Diabetes Care.

[13] Mikolasevic I., Milic S., Orlic L., Poropat G., Jakopcic I., Franjic N., Klanac A., Kristo N., Stimac D. (2016). Metabolic syndrome and acute pancreatitis. Eur. J. Intern. Med..

[14] Vipperla K., Somerville C., Furlan A., Koutroumpakis E., Saul M., Chennat J., Rabinovitz M., Whitcomb D.C., Slivka A., Papachristou G.I. (2017). Clinical Profile and Natural Course in a Large Cohort of Patients with Hypertriglyceridemia and Pancreatitis. J. Clin. Gastroenterol..

[15] Nawaz H., Koutroumpakis E., Easler J., Slivka A., Whitcomb D.C., Singh V.P., Yadav D., Papachristou G.I. (2015). Elevated Serum Triglycerides are Independently Associated with Persistent Organ Failure in Acute Pancreatitis. Am. J. Gastroenterol..

[16] Trikudanathan G., Yazici C., Evans Phillips A., Forsmark C.E. (2024). Diagnosis and Management of Acute Pancreatitis. Gastroenterology.

[17] Tenner S., Vege S.S., Sheth S.G., Sauer B., Yang A., Conwell D.L., Yadlapati R.H., Gardner T.B. (2024). American College of Gastroenterology Guidelines: Management of Acute Pancreatitis. Am. J. Gastroenterol..

[18] He W., Cai W., Yang X., Camilleri G., Zheng X., Wang Q., Li Y., Mukherjee R., Huang W., Sutton R. (2022). Insulin or blood purification treatment for hypertriglyceridaemia-associated acute pancreatitis: A systematic review and meta-analysis. Pancreatology.

[19] Simha V. (2020). Management of hypertriglyceridemia. BMJ.

[20] Yang A.L., McNabb-Baltar J. (2020). Hypertriglyceridemia and acute pancreatitis. Pancreatology.

[21] de Pretis N., Amodio A., Frulloni L. (2018). Hypertriglyceridemic pancreatitis: Epidemiology, pathophysiology and clinical management. United Eur. Gastroenterol. J..

[22] Guo Y.Y., Li H.X., Zhang Y., He W.H. (2019). Hypertriglyceridemia-induced acute pancreatitis: Progress on disease mechanisms and treatment modalities. Discov. Med..

[23] Lu J., Wang Z., Mei W., Peng K., Zhang L., Wang G., Xu K., Wang Z., Peng Y., Lu Z. (2025). A systematic review of the epidemiology and risk factors for severity and recurrence of hypertriglyceridemia-induced acute pancreatitis. BMC Gastroenterol..

[24] Laufs U., Parhofer K.G., Ginsberg H.N., Hegele R.A. (2020). Clinical review on triglycerides. Eur. Heart J..

[25] Scherer J., Singh V.P., Pitchumoni C.S., Yadav D. (2014). Issues in hypertriglyceridemic pancreatitis: An update. J. Clin. Gastroenterol..

[26] Adiamah A., Psaltis E., Crook M., Lobo D.N. (2018). A systematic review of the epidemiology, pathophysiology and current management of hyperlipidaemic pancreatitis. Clin. Nutr..

[27] Fredrickson D.S., Lees R.S. (1965). A System for Phenotyping hyperlipoproteinemia. Circulation.

[28] Fredrickson D.S. (1971). An international classification of hyperlipidemias and hyperlipoproteinemias. Ann. Intern. Med..

[29] Gotoda T., Shirai K., Ohta T., Kobayashi J., Yokoyama S., Oikawa S., Bujo H., Ishibashi S., Arai H., Yamashita S. (2012). Diagnosis and management of type I and type V hyperlipoproteinemia. J. Atheroscler. Thromb..

[30] Brahm A.J., Hegele R.A. (2015). Chylomicronaemia--current diagnosis and future therapies. Nat. Rev. Endocrinol..

[31] Surendran R.P., Visser M.E., Heemelaar S., Wang J., Peter J., Defesche J.C., Kuivenhoven J.A., Hosseini M., Péterfy M., Kastelein J.J. (2012). Mutations in LPL, APOC2, APOA5, GPIHBP1 and LMF1 in patients with severe hypertriglyceridaemia. J. Intern Med..

[32] Okazaki H., Gotoda T., Ogura M., Ishibashi S., Inagaki K., Daida H., Hayashi T., Hori M., Masuda D., Matsuki K. (2021). Current Diagnosis and Management of Primary Chylomicronemia. J. Atheroscler. Thromb..

[33] Brunzell J.D., Bierman E.L. (1982). Chylomicronemia syndrome. Interaction of genetic and acquired hypertriglyceridemia. Med. Clin. N. Am..

[34] Yuan G., Al-Shali K.Z., Hegele R.A. (2007). Hypertriglyceridemia: Its etiology, effects and treatment. CMAJ Can. Med. Assoc. J..

[35] Beigneux A.P., Miyashita K., Ploug M., Blom D.J., Ai M., Linton M.F., Khovidhunkit W., Dufour R., Garg A., McMahon M.A. (2017). Autoantibodies against GPIHBP1 as a Cause of Hypertriglyceridemia. N. Engl. J. Med..

[36] Stroes E.S.G., Alexander V.J., Karwatowska-Prokopczuk E., Hegele R.A., Arca M., Ballantyne C.M., Soran H., Prohaska T.A., Xia S., Ginsberg H.N. (2024). Olezarsen, Acute Pancreatitis, and Familial Chylomicronemia Syndrome. N. Engl. J. Med..

[37] Hegele R.A., Ginsberg H.N., Chapman M.J., Nordestgaard B.G., Kuivenhoven J.A., Averna M., Borén J., Bruckert E., Catapano A.L., Descamps O.S. (2014). The polygenic nature of hypertriglyceridaemia: Implications for definition, diagnosis, and management. Lancet Diabetes Endocrinol..

[38] Brahm A., Hegele R.A. (2013). Hypertriglyceridemia. Nutrients.

[39] Musunuru K., Pirruccello J.P., Do R., Peloso G.M., Guiducci C., Sougnez C., Garimella K.V., Fisher S., Abreu J., Barry A.J. (2010). Exome sequencing, ANGPTL3 mutations, and familial combined hypolipidemia. N. Engl. J. Med..

[40] Klarin D., Damrauer S.M., Cho K., Sun Y.V., Teslovich T.M., Honerlaw J., Gagnon D.R., DuVall S.L., Li J., Peloso G.M. (2018). Genetics of blood lipids among ~300,000 multi-ethnic participants of the Million Veteran Program. Nat. Genet..

[41] Hansen S.E.J., Madsen C.M., Varbo A., Tybjærg-Hansen A., Nordestgaard B.G. (2021). Genetic Variants Associated with Increased Plasma Levels of Triglycerides, via Effects on the Lipoprotein Lipase Pathway, Increase Risk of Acute Pancreatitis. Clin. Gastroenterol. Hepatol..

[42] Guay S.P., Paquette M., Taschereau A., Girard L., Desgagné V., Bouchard L., Bernard S., Baass A. (2024). Acute pancreatitis risk in multifactorial chylomicronemia syndrome depends on the molecular cause of severe hypertriglyceridemia. Atherosclerosis.

[43] FDA FDA Approves Drug to Reduce Triglycerides in Adult Patients with Familial Chylomicronemia Syndrome. https://www.fda.gov/drugs/news-events-human-drugs/fda-approves-drug-reduce-triglycerides-adult-patients-familial-chylomicronemia-syndrome.

[44] Lu Y., George J. (2024). Interaction between fatty acid oxidation and ethanol metabolism in liver. Am. J. Physiol. Gastrointest. Liver Physiol..

[45] Kang L., Sebastian B.M., Pritchard M.T., Pratt B.T., Previs S.F., Nagy L.E. (2007). Chronic ethanol-induced insulin resistance is associated with macrophage infiltration into adipose tissue and altered expression of adipocytokines. Alcohol. Clin. Exp. Res..

[46] Petersen O.H., Tepikin A.V., Gerasimenko J.V., Gerasimenko O.V., Sutton R., Criddle D.N. (2009). Fatty acids, alcohol and fatty acid ethyl esters: Toxic Ca^2+^ signal generation and pancreatitis. Cell Calcium.

[47] Petersen O.H., Sutton R. (2006). Ca^2+^ signalling and pancreatitis: Effects of alcohol, bile and coffee. Trends Pharmacol. Sci..

[48] Huang W., Booth D.M., Cane M.C., Chvanov M., Javed M.A., Elliott V.L., Armstrong J.A., Dingsdale H., Cash N., Li Y. (2014). Fatty acid ethyl ester synthase inhibition ameliorates ethanol-induced Ca^2+^-dependent mitochondrial dysfunction and acute pancreatitis. Gut.

[49] Bessembinders K., Wielders J., van de Wiel A. (2011). Severe hypertriglyceridemia influenced by alcohol (SHIBA). Alcohol Alcohol..

[50] Kahn S.E., Hull R.L., Utzschneider K.M. (2006). Mechanisms linking obesity to insulin resistance and type 2 diabetes. Nature.

[51] Ford E.S., Li C., Zhao G., Pearson W.S., Mokdad A.H. (2009). Hypertriglyceridemia and its pharmacologic treatment among US adults. Arch. Intern. Med..

[52] Tchernof A., Despres J.P. (2013). Pathophysiology of human visceral obesity: An update. Physiol. Rev..

[53] Esparza M.I., Li X., Adams-Huet B., Vasandani C., Vora A., Das S.R., Garg A., Ahmad Z. (2019). Very Severe Hypertriglyceridemia in a Large US County Health Care System: Associated Conditions and Management. J. Endocr. Soc..

[54] Rivellese A.A., De Natale C., Di Marino L., Patti L., Iovine C., Coppola S., Del Prato S., Riccardi G., Annuzzi G. (2004). Exogenous and endogenous postprandial lipid abnormalities in type 2 diabetic patients with optimal blood glucose control and optimal fasting triglyceride levels. J. Clin. Endocrinol. Metab..

[55] Harlow K.E., Africa J.A., Wells A., Belt P.H., Behling C.A., Jain A.K., Molleston J.P., Newton K.P., Rosenthal P., Vos M.B. (2018). Clinically Actionable Hypercholesterolemia and Hypertriglyceridemia in Children with Nonalcoholic Fatty Liver Disease. J. Pediatr..

[56] van de Woestijne A.P., Monajemi H., Kalkhoven E., Visseren F.L. (2011). Adipose tissue dysfunction and hypertriglyceridemia: Mechanisms and management. Obes. Rev..

[57] Subramanian S., Feingold K.R., Adler R.A., Ahmed S.F., Anawalt B., Blackman M.R., Chrousos G., Corpas E., de Herder W.W., Dhatariya K., Dungan K. (2000). Hypertriglyceridemia: Pathophysiology, Role of Genetics, Consequences, and Treatment. Endotext.

[58] Yang Q., Graham T.E., Mody N., Preitner F., Peroni O.D., Zabolotny J.M., Kotani K., Quadro L., Kahn B.B. (2005). Serum retinol binding protein 4 contributes to insulin resistance in obesity and type 2 diabetes. Nature.

[59] Kissebah A.H., Alfarsi S., Evans D.J., Adams P.W. (1982). Integrated regulation of very low density lipoprotein triglyceride and apolipoprotein-B kinetics in non-insulin-dependent diabetes mellitus. Diabetes.

[60] Havel R.J., Gordon R.S. (1960). Idiopathic hyperlipemia: Metabolic studies in an affected family. J. Clin. Investig..

[61] Choi S.H., Ginsberg H.N. (2011). Increased very low density lipoprotein (VLDL) secretion, hepatic steatosis, and insulin resistance. Trends Endocrinol. Metab..

[62] Lan Y.L., Lou J.C., Lyu W., Zhang B. (2019). Update on the synergistic effect of HSL and insulin in the treatment of metabolic disorders. Ther. Adv. Endocrinol. Metab..

[63] Chakrabarti P., Kim J.Y., Singh M., Shin Y.K., Kim J., Kumbrink J., Wu Y., Lee M.J., Kirsch K.H., Fried S.K. (2013). Insulin inhibits lipolysis in adipocytes via the evolutionarily conserved mTORC1-Egr1-ATGL-mediated pathway. Mol. Cell Biol..

[64] Jocken J.W., Langin D., Smit E., Saris W.H., Valle C., Hul G.B., Holm C., Arner P., Blaak E.E. (2007). Adipose triglyceride lipase and hormone-sensitive lipase protein expression is decreased in the obese insulin-resistant state. J. Clin. Endocrinol. Metab..

[65] Sadur C.N., Eckel R.H. (1982). Insulin stimulation of adipose tissue lipoprotein lipase. Use of the euglycemic clamp technique. J. Clin. Investig..

[66] Taskinen M.R., Nikkila E.A. (1979). Lipoprotein lipase activity of adipose tissue and skeletal muscle in insulin-deficient human diabetes. Relation to high-density and very-low-density lipoproteins and response to treatment. Diabetologia.

[67] Nair S., Yadav D., Pitchumoni C.S. (2000). Association of diabetic ketoacidosis and acute pancreatitis: Observations in 100 consecutive episodes of DKA. Am. J. Gastroenterol..

[68] Elkhouly M.A., Salazar M.J., Simons-Linares C.R. (2019). Hypertriglyceridemia-Associated Drug-Induced Acute Pancreatitis. Pancreas.

[69] Herrera E., Ortega-Senovilla H. (2014). Lipid metabolism during pregnancy and its implications for fetal growth. Curr. Pharm. Biotechnol..

[70] Cruciat G., Nemeti G., Goidescu I., Anitan S., Florian A. (2020). Hypertriglyceridemia triggered acute pancreatitis in pregnancy-diagnostic approach, management and follow-up care. Lipids Health Dis..

[71] Hsia S.H., Connelly P.W., Hegele R.A. (1995). Successful outcome in severe pregnancy-associated hyperlipemia: A case report and literature review. Am. J. Med. Sci..

[72] Kumar M.P., Singh A.K., Samanta J., Birda C.L., Kumar N., Dhar J., Gupta P., Kochhar R. (2022). Acute pancreatitis in pregnancy and its impact on the maternal and foetal outcomes: A systematic review. Pancreatology.

[73] Kiss L., Fűr G., Pisipati S., Rajalingamgari P., Ewald N., Singh V., Rakonczay Z. (2023). Mechanisms linking hypertriglyceridemia to acute pancreatitis. Acta Physiol..

[74] Havel R.J. (1969). Pathogenesis, differentiation and management of hypertriglyceridemia. Adv. Intern Med..

[75] Chang Y.T., Chang M.C., Tung C.C., Wei S.C., Wong J.M. (2015). Distinctive roles of unsaturated and saturated fatty acids in hyperlipidemic pancreatitis. World J. Gastroenterol..

[76] Saharia P., Margolis S., Zuidema G.D., Cameron J.L. (1977). Acute pancreatitis with hyperlipemia: Studies with an isolated perfused canine pancreas. Surgery.

[77] Patel K., Durgampudi C., Noel P., Trivedi R.N., de Oliveira C., Singh V.P. (2016). Fatty Acid Ethyl Esters Are Less Toxic Than Their Parent Fatty Acids Generated during Acute Pancreatitis. Am. J. Pathol..

[78] Mateu A., De Dios I., Manso M.A., Ramudo L. (2015). Unsaturated but not saturated fatty acids induce transcriptional regulation of CCL2 in pancreatic acini. A potential role in acute pancreatitis. Biochim. Biophys. Acta.

[79] Noel P., Patel K., Durgampudi C., Trivedi R.N., de Oliveira C., Crowell M.D., Pannala R., Lee K., Brand R., Chennat J. (2016). Peripancreatic fat necrosis worsens acute pancreatitis independent of pancreatic necrosis via unsaturated fatty acids increased in human pancreatic necrosis collections. Gut.

[80] Bai B., Xiang W., Chen X., Chen Q., Liu X., Wang J., Li J., Wang S., Huang J., Gan H. (2026). Oleic acid promotes lung injury in hypertriglyceridaemia-associated acute pancreatitis via the PIEZO1/NR4A1/CPT1A axis impairing endothelial fatty acid oxidation. Gut.

[81] Wen Y., Li Y., Liu T., Huang L., Yao L., Deng D., Luo W., Cai W., Zhong S., Jin T. (2024). Chaiqin chengqi decoction treatment mitigates hypertriglyceridemia-associated acute pancreatitis by modulating liver-mediated glycerophospholipid metabolism. Phytomedicine.

[82] Vollmar B., Menger M.D. (2003). Microcirculatory dysfunction in acute pancreatitis. A new concept of pathogenesis involving vasomotion-associated arteriolar constriction and dilation. Pancreatology.

[83] Qiu M., Zhou X., Zippi M., Goyal H., Basharat Z., Jagielski M., Hong W. (2023). Comprehensive review on the pathogenesis of hypertriglyceridaemia-associated acute pancreatitis. Ann. Med..

[84] Xu T., Sheng L., Guo X., Ding Z. (2022). Free Fatty Acid Increases the Expression of NLRP3-Caspase1 in Adipose Tissue Macrophages in Obese Severe Acute Pancreatitis. Dig. Dis. Sci..

[85] Xia W., Lu Z., Chen W., Zhou J., Zhao Y. (2022). Excess fatty acids induce pancreatic acinar cell pyroptosis through macrophage M1 polarization. BMC Gastroenterol..

[86] Zheng J., Wu J., Chen J., Liu J., Lu Y., Huang C., Hu G., Wang X., Zeng Y. (2016). Therapeutic effects of quercetin on early inflammation in hypertriglyceridemia-related acute pancreatitis and its mechanism. Pancreatology.

[87] Ferrero-Andrés A., Panisello-Roselló A., Roselló-Catafau J., Folch-Puy E. (2020). NLRP3 Inflammasome-Mediated Inflammation in Acute Pancreatitis. Int. J. Mol. Sci..

[88] Xia C.C., Chen H.T., Deng H., Huang Y.T., Xu G.Q. (2024). Reactive oxygen species and oxidative stress in acute pancreatitis: Pathogenesis and new therapeutic interventions. World J. Gastroenterol..

[89] Petersen O.H., Gerasimenko J., Gerasimenko O.V., Gryshchenko O., Peng S. (2021). The Roles of Calcium and ATP in the Physiology and Pathology of the Exocrine Pancreas. Physiol. Rev..

[90] Gerasimenko J.V., Gerasimenko O.V., Petersen O.H. (2014). The role of Ca^2+^ in the pathophysiology of pancreatitis. J. Physiol..

[91] Lee P.J., Papachristou G.I. (2019). New insights into acute pancreatitis. Nat. Rev. Gastroenterol. Hepatol..

[92] Trikudanathan G., Wolbrink D.R.J., van Santvoort H.C., Mallery S., Freeman M., Besselink M.G. (2019). Current Concepts in Severe Acute and Necrotizing Pancreatitis: An Evidence-Based Approach. Gastroenterology.

[93] Cao L., Chen Y., Liu S., Huang W., Wu D., Hong D., Wang Z., Sun Y., Qin K., Guo F. (2023). Early Plasmapheresis Among Patients with Hypertriglyceridemia-Associated Acute Pancreatitis. JAMA Netw. Open.

[94] Banks P.A., Bollen T.L., Dervenis C., Gooszen H.G., Johnson C.D., Sarr M.G., Tsiotos G.G., Vege S.S., Acute Pancreatitis Classification Working G. (2013). Classification of acute pancreatitis--2012: Revision of the Atlanta classification and definitions by international consensus. Gut.

[95] Kiss L., Fűr G., Mátrai P., Hegyi P., Ivány E., Cazacu I.M., Szabó I., Habon T., Alizadeh H., Gyöngyi Z. (2018). The effect of serum triglyceride concentration on the outcome of acute pancreatitis: Systematic review and meta-analysis. Sci. Rep..

[96] Fallat R.W., Vester J.W., Glueck C.J. (1973). Suppression of amylase activity by hypertriglyceridemia. JAMA.

[97] Treacy J., Williams A., Bais R., Willson K., Worthley C., Reece J., Bessell J., Thomas D. (2001). Evaluation of amylase and lipase in the diagnosis of acute pancreatitis. ANZ J. Surg..

[98] Schaefer E.W., Leung A., Kravarusic J., Stone N.J. (2012). Management of severe hypertriglyceridemia in the hospital: A review. J. Hosp. Med..

[99] Pascual I., Sanahuja A., García N., Vázquez P., Moreno O., Tosca J., Peña A., Garayoa A., Lluch P., Mora F. (2019). Association of elevated serum triglyceride levels with a more severe course of acute pancreatitis: Cohort analysis of 1457 patients. Pancreatology.

[100] Deng H., Peng K., Zhang L., Lu J., Mei W., Shi X., Peng Y., Xu K., Li H., Wang Z. (2025). Clinical Outcomes in A Multi-center Cohort Involving 919 Patients with Hypertriglyceridemia-associated Acute Pancreatitis. Am. J. Gastroenterol..

[101] Wang Q., Wang G., Qiu Z., He X., Liu C. (2017). Elevated Serum Triglycerides in the Prognostic Assessment of Acute Pancreatitis: A Systematic Review and Meta-Analysis of Observational Studies. J. Clin. Gastroenterol..

[102] Bálint E.R., Fűr G., Kiss L., Németh D.I., Soós A., Hegyi P., Szakács Z., Tinusz B., Varjú P., Vincze Á. (2020). Assessment of the course of acute pancreatitis in the light of aetiology: A systematic review and meta-analysis. Sci. Rep..

[103] Yang N., Li B., Pan Y., Tu J., Liu G., Lu G., Li W. (2019). Hypertriglyceridaemia delays pancreatic regeneration after acute pancreatitis in mice and patients. Gut.

[104] Yuan S., Giovannucci E.L., Larsson S.C. (2021). Gallstone disease, diabetes, calcium, triglycerides, smoking and alcohol consumption and pancreatitis risk: Mendelian randomization study. npj Genom. Med..

[105] Patel R.S., Pasea L., Soran H., Downie P., Jones R., Hingorani A.D., Neely D., Denaxas S., Hemingway H. (2022). Elevated plasma triglyceride concentration and risk of adverse clinical outcomes in 1.5 million people: A CALIBER linked electronic health record study. Cardiovasc. Diabetol..

[106] Wu B.U., Batech M., Dong E.Y., Duan L., Yadav D., Chen W. (2019). Influence of Ambulatory Triglyceride Levels on Risk of Recurrence in Patients with Hypertriglyceridemic Pancreatitis. Dig. Dis. Sci..

[107] Song K., Wu Z., Meng J., Tian W., Zheng S., Mu D., Wang R., Pang H., Wu D. (2023). Hypertriglyceridemia as a risk factor for complications of acute pancreatitis and the development of a severity prediction model. HPB.

[108] Dong J., Shen Y., Wang Z., Zhang J., Qin X., Zhu C., Gao Y., Yu Q. (2024). Prediction of severe hypertriglyceridemia-associated acute pancreatitis using a nomogram based on CT findings and blood biomarkers. Medicine.

[109] Liu Y., Cheng J.P., Zhao X.L. (2024). The effect of serum triglyceride levels and different lipid-lowering methods on the prognosis of hypertriglyceridemic acute pancreatitis: A single-center 12-year retrospective study by propensity score matching. Scand. J. Gastroenterol..

[110] Hou S., Tang X., Cui H., Liu C., Bai X., Shi L., Shi Y. (2019). Fatty liver disease is associated with the severity of acute pancreatitis: A systematic review and meta-analysis. Int. J. Surg..

[111] Váncsa S., Németh D., Hegyi P., Szakács Z., Hegyi P.J., Pécsi D., Mikó A., Erőss B., Erős A., Pár G. (2020). Fatty Liver Disease and Non-Alcoholic Fatty Liver Disease Worsen the Outcome in Acute Pancreatitis: A Systematic Review and Meta-Analysis. J. Clin. Med..

[112] Chen L., Huang Y., Yu H., Pan K., Zhang Z., Man Y., Hu D. (2021). The association of parameters of body composition and laboratory markers with the severity of hypertriglyceridemia-induced pancreatitis. Lipids Health Dis..

[113] Yang X., Shi N., Yao L., He W., Zhu P., Li S., Li L., Li Y., Liu S., Deng L. (2022). Impact of admission and early persistent stress hyperglycaemia on clinical outcomes in acute pancreatitis. Front. Endocrinol..

[114] Yang X., Zhang R., Jin T., Zhu P., Yao L., Li L., Cai W., Mukherjee R., Du D., Fu X. (2022). Stress Hyperglycemia Is Independently Associated with Persistent Organ Failure in Acute Pancreatitis. Dig. Dis. Sci..

[115] Jin Y., Tao S., Yu G., Li C., Hu Z., Jiang L. (2023). Predictive value of hyperglycemia on infection in critically ill patients with acute pancreatitis. Sci. Rep..

[116] Huang Y., Zhu Y., Peng Y., Xia W., Chen L., Yu H., Shi L., Yang Y., Su N. (2023). Triglycerides to high-density lipoprotein cholesterol (TG/HDL-C) ratio is an independent predictor of the severity of hyperlipidaemic acute pancreatitis. J. Hepatobiliary Pancreat. Sci..

[117] He Y., Wang W., He Q., Ding L., Jin T., Zou K., Deng L., Huang W., Xia Q., Sun X. (2026). Association of metabolic and lipid indices with severity among patients with acute pancreatitis: A 10-year prospective registry-based cohort study with focus on hypertriglyceridemia-associated acute pancreatitis. Clin. Chim. Acta.

[118] Yang L., Liu J., Xing Y., Du L., Chen J., Liu X., Hao J. (2016). Comparison of BISAP, Ranson, MCTSI, and APACHE II in Predicting Severity and Prognoses of Hyperlipidemic Acute Pancreatitis in Chinese Patients. Gastroenterol. Res. Pract..

[119] Shuanglian Y., Huiling Z., Xunting L., Yifang D., Yufen L., Shanshan X., Lijuan S., Yunpeng L. (2023). Establishment and validation of early prediction model for hypertriglyceridemic severe acute pancreatitis. Lipids Health Dis..

[120] Hu J., Sun Y., Hua T., Xiao W., Yang M. (2024). Establishment of Prediction Model for Severe Hypertriglyceridemic Acute Pancreatitis Based on Early Laboratory Indicators. Intensive Care Res..

[121] Wang Z., Liu Y., Zhang X., Wang C., Tian J., Zhao H., Tian Q., Qu H. (2025). Construction of a nomogram for hypertriglyceridemic severe acute pancreatitis that includes metabolic indexes. Lipids Health Dis..

[122] Lu Z., Li M., Guo F., Zhang G., Song S., Liu N., Wang D. (2020). Timely Reduction of Triglyceride Levels Is Associated with Decreased Persistent Organ Failure in Hypertriglyceridemic Pancreatitis. Pancreas.

[123] Leppäniemi A., Tolonen M., Tarasconi A., Segovia-Lohse H., Gamberini E., Kirkpatrick A.W., Ball C.G., Parry N., Sartelli M., Wolbrink D. (2019). 2019 WSES guidelines for the management of severe acute pancreatitis. World J. Emerg. Surg..

[124] De-Madaria E., Buxbaum J.L., Maisonneuve P., Garcia Garcia de Paredes A., Zapater P., Guilabert L., Vaillo-Rocamora A., Rodriguez-Gandia M.A., Donate-Ortega J., Lozada-Hernandez E.E. (2022). Aggressive or Moderate Fluid Resuscitation in Acute Pancreatitis. N. Engl. J. Med..

[125] Al-Ismail M., Promi T., Hetai R., Ahmed S., Awaisu A. (2026). Efficacy and safety of pharmacological and procedural interventions in the management of hypertriglyceridemia-induced acute pancreatitis: A systematic review. Saudi Pharm. J..

[126] Syed-Abdul M.M., Tian L., Hegele R.A., Lewis G.F. (2025). Futility of plasmapheresis, insulin in normoglycaemic individuals, or heparin in the treatment of hypertriglyceridaemia-induced acute pancreatitis. Lancet Diabetes Endocrinol..

[127] Coskun A., Erkan N., Yakan S., Yildirim M., Carti E., Ucar D., Oymaci E. (2015). Treatment of hypertriglyceridemia-induced acute pancreatitis with insulin. Prz. Gastroenterol..

[128] Samad A., James A., Wong J., Mankad P., Whitehouse J., Patel W., Alves-Simoes M., Siriwardena A.K., Bruce J.I. (2014). Insulin protects pancreatic acinar cells from palmitoleic acid-induced cellular injury. J. Biol. Chem..

[129] Mankad P., James A., Siriwardena A.K., Elliott A.C., Bruce J.I. (2012). Insulin protects pancreatic acinar cells from cytosolic calcium overload and inhibition of plasma membrane calcium pump. J. Biol. Chem..

[130] Wu J., Sun Q., Yang H. (2015). Effects of blood glucose control on glucose variability and clinical outcomes in patients with severe acute pancreatitis in intensive care unit. Natl. Med. J. China.

[131] Li J., Chen T.R., Gong H.L., Wan M.H., Chen G.Y., Tang W.F. (2012). Intensive insulin therapy in severe acute pancreatitis: A meta-analysis and systematic review. West Indian Med. J..

[132] Dhindsa S., Sharma A., Al-Khazaali A., Sitaula S., Nadella S., McKee A., Albert S., Bourey R., Dandona P. (2020). Intravenous Insulin Versus Conservative Management in Hypertriglyceridemia-Associated Acute Pancreatitis. J. Endocr. Soc..

[133] Näsström B., Olivecrona G., Olivecrona T., Stegmayr B.G. (2001). Lipoprotein lipase during continuous heparin infusion: Tissue stores become partially depleted. J. Lab Clin. Med..

[134] Weintraub M., Rassin T., Eisenberg S., Ringel Y., Grosskopf I., Iaina A., Charach G., Liron M., Rubinstein A. (1994). Continuous intravenous heparin administration in humans causes a decrease in serum lipolytic activity and accumulation of chylomicrons in circulation. J. Lipid Res..

[135] Wan J., Kuang M., Xiong S., Zou Y., He W., Zhu Y., Lu N., Xia L. (2026). Early administration of low molecular weight heparin in hypertriglyceridemia-induced acute pancreatitis: A propensity score-matched retrospective cohort study. J. Clin. Lipidol..

[136] He W., Ding L., Liu Z., Hua M., Zhou Y., Gong M., Sheng J., Wu X., Fan H., Shu H. (2025). Low-Molecular-Weight Heparin Plus Insulin in Hypertriglyceridemic Acute Pancreatitis: A Randomized Clinical Trial. JAMA Netw. Open.

[137] Gavva C., Sarode R., Agrawal D., Burner J. (2016). Therapeutic plasma exchange for hypertriglyceridemia induced pancreatitis: A rapid and practical approach. Transfus. Apher. Sci..

[138] Click B., Ketchum A.M., Turner R., Whitcomb D.C., Papachristou G.I., Yadav D. (2015). The role of apheresis in hypertriglyceridemia-induced acute pancreatitis: A systematic review. Pancreatology.

[139] Zhang Y., Lin J., Wu L., Lin J., Liang Y. (2022). Blood Purification for Hypertriglyceridemia-Induced Acute Pancreatitis: A Meta-analysis. Pancreas.

[140] Garg R., Rustagi T. (2018). Management of Hypertriglyceridemia Induced Acute Pancreatitis. BioMed Res. Int..

[141] Wang L., Zhou J., Lv C., Hong D., Wang Z., Mao W., Liu Y., Zhang Z., Li Y., Li G. (2024). Impact of therapeutic plasmapheresis on the duration of organ failure in patients with hypertriglyceridemia-associated acute pancreatitis. Ann. Intensive Care.

[142] Zhou J., Wang Z., Liu Q., Cao L., De-Madaria E., Capurso G., Stoppe C., Wu D., Huang W., Chen Y. (2024). Triglyceride-lowering therapies in hypertriglyceridemia-associated acute pancreatitis in China: A multicentre prospective cohort study. BMC Med..

[143] Younossi Z.M., Zelber-Sagi S., Henry L., Gerber L.H. (2023). Lifestyle interventions in nonalcoholic fatty liver disease. Nat. Rev. Gastroenterol. Hepatol..

[144] Gordts P.L., Nock R., Son N.H., Ramms B., Lew I., Gonzales J.C., Thacker B.E., Basu D., Lee R.G., Mullick A.E. (2016). ApoC-III inhibits clearance of triglyceride-rich lipoproteins through LDL family receptors. J. Clin. Investig..

[145] Taskinen M.R., Packard C.J., Borén J. (2019). Emerging Evidence that ApoC-III Inhibitors Provide Novel Options to Reduce the Residual CVD. Curr. Atheroscler. Rep..

[146] Witztum J.L., Gaudet D., Freedman S.D., Alexander V.J., Digenio A., Williams K.R., Yang Q., Hughes S.G., Geary R.S., Arca M. (2019). Volanesorsen and Triglyceride Levels in Familial Chylomicronemia Syndrome. N. Engl. J. Med..

[147] FDA FDA Approves First Treatment Shown to Reduce the Risk of Acute Pancreatitis in Adults with Severe Hypertriglyceridemia. https://www.fda.gov/drugs/news-events-human-drugs/fda-approves-first-treatment-shown-reduce-risk-acute-pancreatitis-adults-severe-hypertriglyceridemia.

[148] Watts G.F., Rosenson R.S., Hegele R.A., Goldberg I.J., Gallo A., Mertens A., Baass A., Zhou R., Muhsin M., Hellawell J. (2025). Plozasiran for Managing Persistent Chylomicronemia and Pancreatitis Risk. N. Engl. J. Med..

[149] Gaudet D., Pall D., Watts G.F., Nicholls S.J., Rosenson R.S., Modesto K., San Martin J., Hellawell J., Ballantyne C.M. (2024). Plozasiran (ARO-APOC3) for Severe Hypertriglyceridemia: The SHASTA-2 Randomized Clinical Trial. JAMA Cardiol..

[150] Rosenson R.S., Gaudet D., Ballantyne C.M., Baum S.J., Bergeron J., Kershaw E.E., Moriarty P.M., Rubba P., Whitcomb D.C., Banerjee P. (2023). Evinacumab in severe hypertriglyceridemia with or without lipoprotein lipase pathway mutations: A phase 2 randomized trial. Nat. Med..

[151] Watts G.F., Schwabe C., Scott R., Gladding P.A., Sullivan D., Baker J., Clifton P., Hamilton J., Given B., Melquist S. (2023). RNA interference targeting ANGPTL3 for triglyceride and cholesterol lowering: Phase 1 basket trial cohorts. Nat. Med..

[152] Gaudet D., Gonciarz M., Shen X., Leohr J.K., Beyer T.P., Day J.W., Mullins G.R., Zhen E.Y., Hartley M., Larouche M. (2025). Targeting the angiopoietin-like protein 3/8 complex with a monoclonal antibody in patients with mixed hyperlipidemia: A phase 1 trial. Nat. Med..

[153] Raal F.J., Rosenson R.S., Reeskamp L.F., Hovingh G.K., Kastelein J.J.P., Rubba P., Ali S., Banerjee P., Chan K.C., Gipe D.A. (2020). Evinacumab for Homozygous Familial Hypercholesterolemia. N. Engl. J. Med..

[154] Bergmark B.A., Marston N.A., Bramson C.R., Curto M., Ramos V., Jevne A., Kuder J.F., Park J.G., Murphy S.A., Verma S. (2022). Effect of Vupanorsen on Non-High-Density Lipoprotein Cholesterol Levels in Statin-Treated Patients with Elevated Cholesterol: TRANSLATE-TIMI 70. Circulation.

[155] Oostveen R.F., Hovingh G.K., Stroes E.S.G. (2023). Angiopoietin-like 3 inhibition and the liver: Less is more?. Curr. Opin. Lipidol..

[156] Jung K.H., Son M.K., Yan H.H., Fang Z., Kim J., Kim S.J., Park J.H., Lee J.E., Yoon Y.C., Seo M.S. (2020). ANGPTL4 exacerbates pancreatitis by augmenting acinar cell injury through upregulation of C5a. EMBO Mol. Med..

[157] Kersten S. (2021). Role and mechanism of the action of angiopoietin-like protein ANGPTL4 in plasma lipid metabolism. J. Lipid Res..

[158] Dewey F.E., Gusarova V., O’Dushlaine C., Gottesman O., Trejos J., Hunt C., Van Hout C.V., Habegger L., Buckler D., Lai K.M. (2016). Inactivating Variants in ANGPTL4 and Risk of Coronary Artery Disease. N. Engl. J. Med..

[159] Chen Y.Q., Pottanat T.G., Siegel R.W., Ehsani M., Qian Y.W., Zhen E.Y., Regmi A., Roell W.C., Guo H., Luo M.J. (2020). Angiopoietin-like protein 8 differentially regulates ANGPTL3 and ANGPTL4 during postprandial partitioning of fatty acids. J. Lipid Res..

